# Barriers for work in people with multiple sclerosis: a Norwegian cultural adaptation and validation of the short version of the multiple sclerosis work difficulties questionnaire

**DOI:** 10.3389/fresc.2024.1404723

**Published:** 2024-11-20

**Authors:** Britt Normann, Ellen Christin Arntzen, Cynthia A. Honan

**Affiliations:** ^1^ Faculty of Nursing and Health Science, Nord University, Bodø, Norway; ^2^ Department of Physiotherapy, Nordland Hospital Trust, Bodø, Norway; ^3^ Department of Physiotherapy, Kongsgården Physiotherapy A/S, Bodø, Norway; ^4^ School of Psychological Sciences, University of Tasmania, Launceston, TAS, Australia; ^5^ Launceston General Hospital, Launceston, TAS, Australia

**Keywords:** multiple sclerosis, work, employment status, validation studies, surveys, questionnaires

## Abstract

**Background and purpose:**

Multiple sclerosis (MS) is associated with high rates of unemployment, and barriers for work are essential to identify in the regular follow-up of these people. The current study aimed to culturally adapt and evaluate the psychometric properties of the Norwegian version of the Multiple Sclerosis Work Difficulties Questionnaire-23 (MSWDQ-23).

**Methods:**

Following backward and forward translation, the Norwegian version of the MSWDQ-23 (MSWDQ-23NV) was completed by 229 people with multiple sclerosis (MS). Validity was evaluated through confirmatory factor analysis and by associating scores with employment status, disability, and health-related quality of life outcome measures. Convergent validity was checked by correlating MSWDQ-23 scores with alternative study measures. Internal consistencies were examined by Cronbach's alfa.

**Results:**

A good fit for the data was demonstrated for the MSWDQ-23NV in confirmatory factor analysis, with excellent internal consistencies also demonstrated for the full scale and its subscales (physical barriers, psychological/cognitive barriers, external barriers). The MSWDQ-23NV subscales were related in the expected direction to health-related quality of life outcome measures. While higher scores on the physical barriers subscale was strongly associated with higher levels of disability and progressive MS types, higher scores on all subscales were associated with not working in the past year.

**Discussion:**

The Norwegian MSWDQ-23 is an internally consistent and valid instrument to measure perceived work difficulties in persons with all types of MS in a Norwegian-speaking population. The MSWDQ-23NV can be considered a useful tool for health care professionals to assess self-reported work difficulties in persons with MS. The Norwegian MSWDQ-23 scale should be examined for test-retest reliability and considered implemented in the regular follow up at the MS-outpatient clinics in Norway to support employment maintenance.

## 1 Introduction

Multiple Sclerosis (MS) is a chronic immune-mediated, demyelinating degenerative disease of the central nervous system affecting young adults of working age (1). A variety of symptoms may occur, such as visual and sensorimotor disturbances, cognitive difficulties, pain and fatigue (1). The disease is associated with early retirement from work and high rates of unemployment (2–5), which in turn reduces quality of life (6), and increase personal burdens and societal economic costs (3–5, 7–9). Norway is a high risk area for MS with a prevalence of 248/100 000 and incidence 8–12/100 000 (10). In the Norwegian population, minor to moderate levels of disability, measured by the Expanded Disability Status Scale (EDSS), dominates (10, 11). Still, unemployment is reported in 55%–70% of Norwegian people with MS (pwMS) (7, 12, 13). Globally, 43% of pwMS quit their job within the first 3 years after diagnosis, and 70% quit within 10 years (4).

There is little monitoring and follow-up of employment difficulties or the need for work adaptations at hospitals’ MS-outpatient (MS-OP) clinics. Consequently, employment status, and known work barriers such as fatigue, poor mobility and physical functioning, cognitive difficulties, depression, anxiety, and difficulties balancing the workload and family-life (12, 13, 14–17) are not typically addressed and remediated. Immense prospects for personal and societal benefits exist if employment issues are addressed early at routine follow-up of pwMS. One issue preventing the successful identification of work difficulties in pwMS at routine follow-up, however, is the lack standardized Norwegian instruments available to assess these difficulties.

Multiple Sclerosis Work Difficulties Questionnaire (MSWDQ), developed by Honan et al. is a suitable self-report instrument, developed to assess the perceived impact of MS on work difficulties (18). Because of its length (50 items), the authors subsequently created a shortened version (MSWDQ-23 containing 23 items), which was more suited for clinical practice (19). Both the full and shortened versions show good psychometric properties (18, 19) and the MSWDQ-23 has been translated into several European languages (20–26) as well as Turkish, Tunisian and Persian (27–29). The measure has also been successfully used to predict and understand a range of negative work outcomes. For example, the work difficulties assessed in the MSWDQ-23 have not only consistently been found to be related to withdrawal from work and productivity loss (22, 30), but they have also been found to mediate the relationship between job demands and burnout, between job resources and work engagement, and between job resources and burnout (31). The MSWDQ-23 contains 23 items with five response options [0 (never), 1 (rarely), 2 (sometimes), 3 (often) and 4 (almost always)], which assess how frequently the individual experienced difficulties over the previous four weeks in their current or most recent job. Items are grouped into three subscales (physical, psychological/cognitive, and external barriers). Patients’ perception of psychological and cognitive barriers in the workplace, as measured by the MSWDQ-23, is predictive of both unemployment and reducing the number of work hours since the MS diagnosis (32). A Norwegian version of the MSWDQ-23 may be an adequate tool to assess work difficulties in the Norwegian MS population and to address the specific work-related challenges that occur within this group.

The current study aims to culturally adapt the MSWDQ-23 for the Norwegian population and to examine its validity by verifying its three-factor structure and evaluating its other psychometric properties. We hypothesized that the Norwegian version of MSWDQ-23 would demonstrate acceptable internal consistency and model of fit based on confirmatory factor analysis, in line with previous validation studies (19, 22). We also expected acceptable construct validity with conceptually related measures of physical disability, psychological and cognitive difficulties, and health-related quality of life, using defined hypothesis.

## 2 Materials and methods

The study consists of two parts. (I) The translation and cultural adaptation. (II) The validation study.

### 2.1 Ethics

The study was approved by the Reginal Committee for Medical and Health Research Ethics, Norway (REK Nord 174837) and the Nordland Hospital Research Ethical Authority (project number 209). We conducted in accordance with the Helsinki declaration, and all participants provided written informed consent prior to inclusion. The study was funded by the Nortern Norway Regional Health Authority (Grant number 174837). The funder played no role in the design, conduct or reporting of the study.

### 2.2 Part I

The adaptation of the MSWDQ-23 (19) into Norwegian, followed the recommendations made by the International Society for Pharmacoeconomics and Outcomes Research (33), containing nine steps, described in the following.

**Figure 1:**
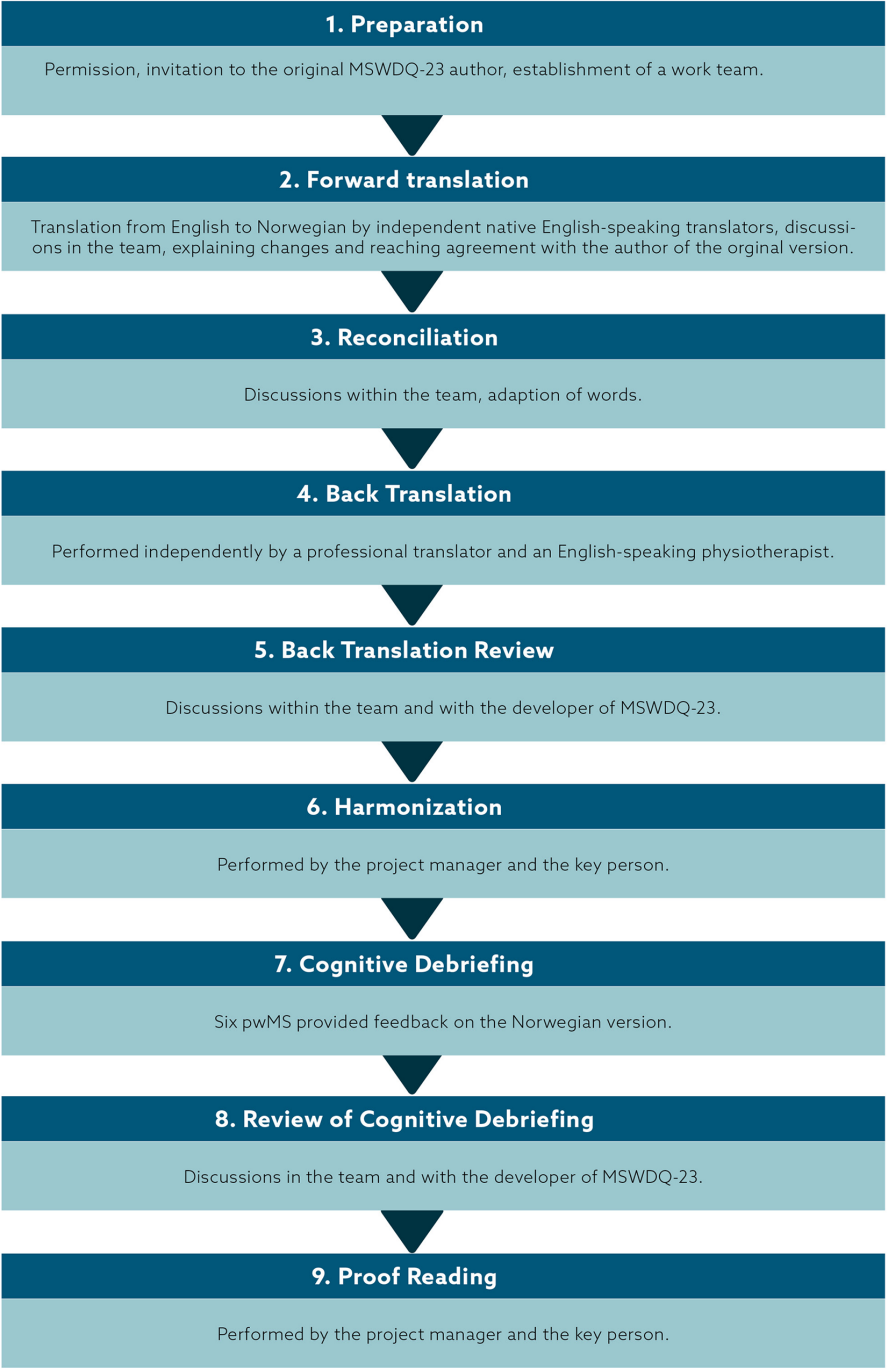
Process and performance indicators for translation and adaptation of the MSWDQ-23.

(1) First, we obtained permission from the developer of MSWDQ-23 who agreed to participate as a consultant. Then we established a team consisting of one key person (ECA) who worked closely with the project manager (BN) throughout the adaptation process, two native Norwegian physical therapists to conduct the forward translation of whom one had studied in English speaking countries. For the back translation, we recruited one other native Norwegian physical therapist, who had studied in English speaking countries, and one native English speaker who was also fluent in Norwegian and worked as a professional translator. (2) The team members independently translated the questionnaire into Norwegian and made comments regarding ambiguities or word choice. (3) Reconciliation: The team members discussed the translated drafts and recognized some variations and challenges. The Norwegian version introduced some language changes in the instructions and some of the items. In Norwegian the word “please” is not so commonly used while giving instructions. We decided, in line with the Spanish version, to preserve the word. However, we did not repeat it twice in the last sentence of the introduction. When translating “I found” and “I thought” we used the same Norwegian word (“jeg syntes”). We used job (“jobb”) instead of work and leader (“leder”) instead of manager. Moreover, we discussed the differences between “I thought”, “I felt”, “I experienced”, “I found”, “I feared” with the original MSWDQ author (CH) to ensure that the translation covered the intended meaning. Since the Norwegian health care system implies that there is no immediate loss of payment, we, in agreement with CH, changed item No.21 (corresponding No.48 in the original 50-item scale) to include future decrease of payment. (4) By involving both a professional with knowledge in MS and experience of treating these patients, as well as a native English person with professional linguistic knowledge in the back translation of the reconciled translation of the questionnaire, we aimed for both conceptual, cultural as well as linguistic equivalence. (5) Back translation review was first discussed within the team and then with the developer of the original MSWDQ-23 (CH). (6) Harmonization was conducted by the project manager (BN) and the key person (ECA) by comparing the original, the Norwegian version and the back translated version. (7) Cognitive debriefing was obtained by distributing the Norwegian version of MSWDQ-23 to a group of pwMS (*n* = 6) who completed the questionnaire and were telephone interviewed about their item interpretation/understanding. (8) The whole team reviewed the cognitive debriefing results and made some adjustments. (9) Proofreading was performed by the BN and ECA. An overview of the process is shown in Figure 1. The MSWDQ-23 Norwegian Version (MSWDQ-23NV) is available as Supplementary Materials.

### 2.3 Part II

#### 2.3.1 Design

A non-interventional, cross-sectional validation study was carried out in Nordland County, Norway from July 2021–January 2022.

#### 2.3.2 Participants

Study participants were recruited in Nordland County, through the Nordland Hospital Trust. Inclusion criteria were: diagnosed with MS according the McDonald's criteria (34) and ≥18–67 years of age. There were no exclusion criteria. There were 252 pwMS who originally participated in the study. Among these, 23 participants were removed due to excessive missing data (>50% on the MSWDQ-23NV) (19), leaving 229 participants. The demographic characteristics of the original and final sample of participants are presented in Table 1. Most participants in the final sample had relapsing remitting MS (RRMS) and were female, and more than half were in paid employment within the past year. Most participants completed either a bachelor's degree or high school education. The average age of the participants was 51.6 (SD 12.5) years and the average EDSS was 2.8 (SD 2.2). The final sample of participants represented individuals from 45 municipalities across Nordland County, with the majority from Bodø (24.5%) and Rana (13.5%) municipalities.

**Table 1:** Participant demographic information and disease characteristics.

Characteristics	Original sample (*N* = 252)	Final sample (*N* = 229)
Age, *M* (SD)	52.35 (12.88)	51.56 (12.52)
Female, *n* (%)	177 (70.24)	166 (72.49)
Time since diagnosis (years)	13.18 (10.00) Missing 1	12.54 (9.33)
MS subtype, *n* (%)		
Relapsing-remitting MS	177 (70.24)	169 (73.80)
Secondary-progressive MS	34 (13.49)	25 (10.92)
Primary-progressive MS	34 (13.49)	29 (12.66)
Missing	7 (2.78)	6 (2.62)
EDSS	2.81 (2.19) Missing 8 (3.17)	2.62 (2.07) Missing 7 (3.06)
Currently employed, *n* (%)		
Yes	144 (57.14)	136 (59.39)
No	108 (42.86)	93 (40.61)
Worked in previous year, *n* (%)		
Yes	126 (50.00)	126 (55.02)
No	126 (50.00)	103 (44.98)
Relationship status, *n* (%)		
Married	114 (45.24)	106 (46.29)
Partner	61 (24.21)	56 (24.45)
Single	75 (29.76)	65 (28.38)
Missing	2 (0.79)	2 (0.87)
Highest level of education, *n* (%)		
Did not complete High School	43 (17.06)	31 (13.54)
High School	93 (36.90)	89 (38.86)
Bachelor's degree	87 (34.52)	82 (35.81)
Master's degree	22 (8.73)	22 (9.61)
Doctoral degree	1 (0.40)	1 (0.44)
Missing	6 (2.38)	4 (1.75)
Height, *M* (SD)	170.67 (8.22)	170.67 (8.22)
Weight, *M* (SD)	78.32 (17.44)	78.32 (17.45)
MSQoL54, *M* (SD)		
Physical function	64.68 (30.19)	67.00 (29.16)
Role limitations-physical	38.65 (42.08)	39.74 (42.13)
Role limitations-emotional	66.40 (41.42)	67.54 (41.17)
Pain	67.01 (26.05)	67.05 (26.16)
Emotional well-being	73.87 (17.01)	73.73 (17.16)
Energy	42.38 (22.25)	42.22 (22.48)
Health perceptions	51.53 (22.44)	52.29 (22.54)
Social function	71.78 (22.26)	72.05 (22.02)
Cognitive function	70.95 (22.64)	70.46 (22.86)
Health distress	69.96 (24.14)	69.89 (24.09)
Sexual function	65.38 (28.48)	65.98 (27.90)
Physical health composite	59.47 (19.69)	59.20 (19.41)
Mental health composite	69.73 (18.90)	70.03 (19.21)
Overall quality of life	66.67 (17.51)	67.14 (17.66)

#### 2.3.3 Procedure

Informed consent forms and the questionnaires were sent by mail to all persons with MS who were registered at Nordland Hospital (NLSH) (512 persons). After two reminders, 252 persons had provided their written informed consent, and had returned their completed questionnaires to the MS-outpatient clinic at NLSH.

#### 2.3.4 Measures

To record perceived work difficulties, the Norwegian version of MSWDQ-23 (19) was administered. Subscale scores regarding physical, psychological/cognitive, and external barriers, were computed by summing the observed items scores and dividing this by the total possible item scores in each subscale and then multiplying the value by 100. The maximum score for each dimension is 100, with higher scores indicating higher perceived difficulties. The total MSWDQ-23 score is the average of the three subscale scores.

##### 2.3.4.1 Expanded disability status scale

The Expanded Disability Status Scale (EDSS) is a method of quantifying disability in multiple sclerosis (35). The EDSS scale ranges from 0 to 10 with 0.5-unit increments that represent higher levels of disability. Scoring is based on an examination by a neurologist. It is widely used in clinical trials and in the assessment of pwMS.

##### 2.3.4.2 Multiple sclerosis quality of life-54

Multiple Sclerosis Quality of Life-54 (MSQOL-54) (36) measures health related quality of life. It is a validated instrument with an adequate test-retest reliability, construct validity and internal consistency (37). MSQOL-54 contains 52 QOL items that are divided across 12 scales (physical function, role limitations-physical, role limitations-emotional, pain, emotional well-being, energy, health perceptions, social function, cognitive function, health distress, overall quality of life, and sexual function) and two single items (satisfaction with sexual function and change in health) (37). Two summary scores, physical health composite score (PHCS) and mental health composite score (MHCS) are derived from a weighted combination of scale scores. The composite scores range from 0 to100, with a higher score indicating a better perceived health.

#### 2.3.5 Demographic and employment registrations

In the survey the participants registered their age, time since diagnosis, type of MS, EDSS value, height, weight, relationship status, level of education, their work% the previous year, % of disability benefits, sick leave, and recovery benefits and how much they wanted to work if the job had been perfectly adjusted to their needs.

#### 2.3.6 Statistical analyses

The three-factor structure of the MSWDQ-23 as originally devised by Honan et al. (19), was examined using Confirmatory Factor Analysis (CFA) in AMOS (Version 27.0) (38). Since CFA assumes that variables are normally distributed, in accordance with the recommendations of Enders, the Bollen-Stine bootstrapping procedure was used (39, 40). Positively skewed distributions were present in (1) the following psychological/cognitive barriers subscale: “colleagues not supportive”, “needed to be reminded”, “interact with people”, and “forgot what task”; (2) the following physical barriers subscale: “tolerate temperature”, “accessing office/worksite”, “experienced pain”, “write or type”, and “feared incontinent”; and (3) in the following external barriers subscale: “responsibilities at home”. Acceptable fit was evaluated against the recommendations of Hu and Bentler (41) and Kline (42). According to Hu and Bentler a normed *χ^2^* (*χ^2^/df*) <2 indicates good model fit, a root-mean squared error of approximation (RMSEA) of <.08 indicates fair model fit (90% confidence intervals of ≤.05 and ≤.10 for the lower and upper bound, respectively, indicates good model fit), a comparative fit index (CFI) >.90 indicates fair model fit (>.95 indicates good fit), and a standardized root-mean-square residual (SRMR) <.08 indicates good model fit. According to Kline's less conservative criteria, acceptable model fit is indicated by a *χ^2^/df* <3, the lower-bound of the 90% RMSEA ≤.05 and upper bound ≤.10, a CFI >.90, and SRMR <.10. Bayesian information criteria (BIC) values were also interpreted with lower numbers representative of better fit. To better reflect the structure of the MSWDQ-23 in participants experiencing work difficulties and to improve factorability of the data, 16 cases with extremely low scores (<3) were removed prior to the CFA. Missing data for the MSWDQ-23 items on the sample of 229 participants was minimal (≤2.2%), and the data was missing completely at random (Little's MCAR test, *χ^2^* = 409.94, df = 384, *p* = .174). The missing data was estimated using the expectation maximization method in SPSS prior to the CFA and validity analyses.

Floor and ceiling effects were calculated on the 229 participants. These effects were considered present if 15% of the participants achieved the highest or lowest score possible on the total MSWDQ-23 scale or its subscales (22). Internal consistency of the subscales was examined using Cronbach's α, with values ≥.70 and ≥.80 representing acceptable and excellent internal consistencies, respectively (43).

Construct validity of the MSWDQ-23 was examined using Pearson correlation analyses with alternative subscales included in the study (i.e., the MSQoL-54) and between the MSWDQ-23 subscales (i.e., factorial validity, a form of construct validity).The interpretation of correlations were guided by the recommendations of Cohen (44), where .10, .30, and .50 indicate small, moderate, and large effects, respectively. The relative magnitude of the correlations was used to establish convergence or divergence with alternative measures, with those at least moderate in size generally deemed to establish convergence. The concurrent validity of the MSWDQ-23 was examined using independent samples *t*-tests (one-tailed due to *a priori* directional hypothesis) for employment status, one-way analysis of variance (ANOVA) with Sidak correction to control for inflated alpha for MS type. IBM SPSS Version 28 (45) was used to conduct the analyses. Assumptions for these analyses were met, except where specified in the Results section.

## 3 Results

### 3.1 Floor and ceiling effects

No floor effects with the best possible score of zero on the MSWDQ-23 were present (5.2% of participants had scores of 0 and no participant had scores of 100). For the individual subscales, while no floor effects were present with the psychological/cognitive and physical barriers subscales (8.7% and 8.3%, respectively with best possible scores) a floor effect was present in the external barriers subscale, with 15.3% of participants indicating the best possible score of zero. There were no ceiling effects present in the total MSWDQ-23 or subscales.

### 3.2 Confirmatory factor analysis

An initial CFA produced the following statistics: *χ^2^/df* = 3.016, RMSEA = .098 (lower bound = .089, upper bound = .106), SRMR = 0.0736, CFI = 0.809, and BIC = 846.96, indicating a poor fit of the data. Following inspection of the modification indices, several conceptually linked error terms were correlated (19, 22). In the psychological/cognitive barriers subscale these included: “forgot what task”, “needed to be reminded” and “remember conversation”; and “employer not understanding” and “colleagues not supportive”. In the physical barriers subscale these included: “disturbances in bowel/bladder” and “feared incontinent”. In the external barriers subscale these included: “feared no longer support self” and “reduce work hours, reduced pay”. Correlating the error terms improved the model fit as follows: *χ^2^/df* = 1.979, RMSEA = .068 (lower bound = .059, upper bound = .077), SRMR = 0.0692, CFI = 0.910, and BIC = 613.347. These indices represent overall fair model fit for the data, thus supporting the structure of the MSWDQ-23. Modification indices pertaining to item loadings were also inspected to see if any removal would improve fit indices, particularly the lower bound of the RMSEA which remained slightly outside the requirements for *good* fit. The removal of items “perform to level expected” and “became sleepy” in the psychological/cognitive barriers subscale improved the fit further as follows: *χ^2^/df* = 1.796, RMSEA = .061 (lower bound = .050, upper bound = .072), SRMR = 0.0686, CFI = 0.931, and BIC = 483.957. However, given the very high correlation (*r* = .988) between the scores for the psychological/cognitive barriers subscale with and without these items, this improvement was determined to be negligible, hence supporting the inclusion of all 23 items in the scale. Table 2 shows factor loadings for the MSWDQ-23.

**Table 2:** Factor loadings and internal reliability of the Norwegian MSWDQ-23 (*N* *=* 229).

	Factor loading	Corrected item-total correlation	Cronbach's alpha if item deleted
Psychological/cognitive barriers (Cronbach's *α* = .91)			
Item 2 “employer not understanding”	.45	.52	.91
Item 3 “learn something new”	.64	.66	.90
Item 4 “colleagues not supportive”	.42	.50	.91
Item 6 “needed to be reminded”	.62	.62	.91
Item 7 “perform to level expected”	.69	.70	.90
Item 10 “remember conversation”	.70	.71	.90
Item 13 “became sleepy”	.71	.67	.90
Item 15 “concentrating”	.83	.80	.90
Item 16 “communicating thoughts”	.75	.74	.90
Item 19 “interact with people”	.76	.74	.90
Item 22 “forgot what task”	.74	.72	.90
Physical barriers (Cronbach's *α* = .84)			
Item 1 “lack of coordination in movements”	.74	.67	.80
Item 5 “disturbances in bowel/bladder”	.56	.60	.82
Item 8 “tolerate temperature”	.35	.36	.84
Item 9 “accessing office/worksite”	.59	.53	.82
Item 11 “experienced pain”	.64	.60	.82
Item 14 “maintain balance”	.74	.71	.80
Item 18 “write or type”	.57	.52	.83
Item 20 “feared incontinent”	.52	.56	.82
External barriers (Cronbach's *α* = .83)			
Item 12 “feared no longer support self”	.57	.62	.81
Item 17 “balance work and home duties”	.83	.69	.77
Item 21 “reduced work hours, reduced pay”	.62	.70	.77
Item 23 “responsibilities at home”	.78	.64	.80

Note: Please see Honan et al. (18, 19) for item questions. All loadings were significant to the *p* < .001 level. MSWDQ-23, multiple sclerosis work difficulties questionnaire-23.

Correlations between the three subscales were large (psychological/cognitive barriers and physical barriers, *r* = .611; psychological/cognitive barriers and external barriers, *r* = .846; and physical barriers and external barriers, *r* = .596). Given the very large correlation between the psychological/cognitive barriers and external barriers, an alternative model with respective items for these subscales loading onto a single factor was conducted in CFA. However, this did not improve the fit of the model [*χ^2^/df* = 2.119, RMSEA = .073 (lower bound = .064, upper bound = .082), SRMR = 0.0692, CFI = 0.896, and BIC = 643.955].

A second-order CFA with all 23 items loading onto the three factors, and in turn, a further higher-order overall MSWDQ factor was also examined. Fit statistics for this model were identical to the first order model and were as follows: *χ^2^/df* = 1.979, RMSEA = .068 (lower bound = .059, upper bound = .077), SRMR = 0.0692, CFI = 0.910, and BIC = 613.347.

### 3.3 Internal consistency

Cronbach's α values for the MSWDQ-23 subscales are shown in Table 2. All subscales had excellent internal consistency. No removal of any item would substantially improve these internal consistency values. Cronbach's α was.94 for the full MSWDQ-23.

### 3.4 Construct validity

The Norwegian MSWDQ-23 physical, psychological/cognitive, and external barriers subscales, had large, small-to-moderate, and small positive relationships respectively with EDSS scores (46). Total MSWDQ-23 scores were moderately and positively related to EDSS. There were large relationships present between the MSWDQ-23 total and subscales and the physical and the mental health composite scores. There were moderate-to-large relationships present between Total MSWDQ-23 and psychological/cognitive barriers scores and employment status. Physical barriers and external barriers had a moderate and small relationship respectively with work status (%). Table 3 shows correlations with the alternative scales used in this study.

**Table 3:** Construct validity: Pearson correlations between MSWDQ-23 total and subscales correlations with alternative measures (*N* = 229).

	Total MSWDQ-23	Psychological/Cognitive barriers	Physical barriers	External barriers
EDSS	**.** **25**	.14	.**48**	.03
Multiple sclerosis quality of life-54				
Physical function	**-0**.**33**	**−0**.**21**	**−0**.**52**	−0.16
Role limitations-physical	**−0**.**43**	**−0**.**36**	**−0**.**44**	**−0**.**35**
Role limitations-emotional	**−0**.**37**	**−0**.**36**	**−0**.**30**	**−0**.**29**
Pain	**−0**.**42**	**−0**.**30**	**−0**.**53**	**−0**.**31**
Emotional well-being	**−0**.**45**	**−0**.**44**	**−0**.**37**	**−0**.**36**
Energy	**−0**.**52**	**−0**.**50**	**−0**.**38**	**−0**.**51**
Health perceptions	**−0**.**50**	**−0**.**41**	**−0**.**49**	**−0**.**46**
Social function	**−0**.**50**	**−0**.**45**	**−0**.**49**	**−0**.**39**
Cognitive function	**−0**.**64**	**−0**.**69**	**−0**.**41**	**−0**.**55**
Health distress	**−0**.**50**	**−0**.**44**	**−0**.**46**	**−0**.**43**
Sexual function	**−0**.**20**	−0.14	**−0**.**26**	−0.11
Physical health composite	**−0**.**59**	**−0**.**48**	**−0**.**63**	**−0**.**47**
Mental health composite	**−0**.**59**	**−0**.**57**	**−0**.**49**	**−0**.**49**
Overall quality of life	**−0**.**49**	**−0**.**42**	**−0**.**48**	**−0**.**41**

Note: EDSS, expanded disability status scale; MSWDQ-23, multiple sclerosis work difficulties questionnaire-23.

Bolded correlations were significant at the *p* < 0.0 (2-tailed) level.

### 3.5 Concurrent validity

MSWDQ total and subscale scores according to MS type, together with inferential statistical information, is shown in Table 4. While differences in MSWDQ-23 scores were not detected in the psychological/cognitive barriers and external barriers subscales, and the full MSWDQ-23, group differences were found in the physical barriers subscale. In particular, the participants with RRMS had significantly fewer physical work difficulties than both the SPMS and PPMS participants. There were no differences between SPMS and PPMS in physical barriers scores. MSWDQ total and subscale scores according to employment status, together with inferential statistic information, is shown in Table 5. The participants who were working over the previous 12 months had significantly fewer work difficulties than people who had not been working over the previous 12 months.

**Table 4:** Descriptive and inferential statistics for the MSWDQ-23 stratified by MS type (*N* = 223).

Subscales	Overall *N*	MS type				
		RRMS	SPMS	PPMS	F-score	*p*-value
Psychological/cognitive barriers	30.18 (21.19)	29.73 (21.66)	31.83 (19.50)	31.34 (20.36)	.156	.856
Physical barriers	29.47 (20.90)	26.17 (20.25)^a,b^	40.94 (18.18)^a^	38.81 (21.12)^b^	9.44	<.001
External barriers	39.08 (28.26)	39.42 (29.09)	40.63 (27.10)	35.76 (24.71)	.247	.782
Total MSWDQ-23	31.48 (19.78)	30.18 (20.17)	36.53 (17.58)	34.71 (18.76)	1.58	.209

Note: Means are provided with standard deviations in brackets. Six cases were not included in this analysis due to missing data on MS type. Multiple Sclerosis Work Difficulties Questionnaire-23; PPMS, primary-progressive MS; RRMS, relapsing-remitting MS; SPMS, secondary-progressive MS.

^a^ *p* < .01 for difference between RRMS and SPMS.

^b^ *p* < .01 for difference between RRMS and PPMS.

**Table 5:** Descriptive and inferential statistics for the MSWDQ-23 stratified by employment status (*N* = 229).

Subscales	Employment status			
	Have worked in the past year	Have not worked in the past year	*t*-score	*p*-value
Psychological/cognitive barriers	26.34 (19.53)	34.47 (21.97)	2.96	.002
Physical barriers	22.70 (17.96)	37.09 (21.28)^a^	5.55	<.001
External barriers	35.75 (27.10)	41.98 (29.12)	1.67	.048
Total MSWDQ-23	26.71 (17.49)	36.68 (20.80)^a^	1.58	<.001

Note: Means are provided with standard deviations in brackets. One-tailed probability values are reported. Multiple Sclerosis Work Difficulties Questionnaire-23.

^a^ Equal variances not assumed statistics reported.

## 4 Discussion

The goal of the current study was to culturally adapt the MSWDQ-23 for the Norwegian population and to verify the three-factor structure and evaluate the psychometric properties of the Norwegian version of the MSWDQ-23. The Norwegian version of the MSWDQ-23 and its subscales showed an excellent internal consistency, similar to the original questionnaire (19) and previous validation studies (21–24, 27, 28). It may be concluded that the original 3-factor structure of the MSWDQ-23 are acceptable in the Norwegian-translated version of the scale. However, the fit of the data in the CFA would also suggest that the overall MSWDQ-23NV total scores are also interpretable. In addition, given the high correlation between the scales psychological/cognitive barriers and external barriers, and support for adequate model fit, these scales may be combined and interpreted together in the Norwegian Version.

It seems that in the Norwegian population, psychological and cognitive barriers are very much tied to external barriers. The causal direction of this relationship, however, is not known. In the Australian study (19), these correlation between these barriers was only .72 compared to.85 in the Norwegian study. Note that the prior Dutch-language validation study also exhibited high correlations, but the pattern of the relationships were slightly different (22). They had quite large relationships between physical and psychological (.84) and between physical and external (.91) barriers. Psychological and external barriers was correlated .76. All these relationships, nonetheless, are likely to reflect sampling differences rather than regional/population differences. At the end of the day, all subscales are very much related to each other regardless of region/population.

No ceiling effects were present in the total MSWDQ-23NV or subscales. There were floor effects present in the external barriers subscale, although scores for this subscale were on average higher than the other two subscales. The floor effect may reflect the possibility of a proportion of pwMS in the sample who are accessing benefits and the strong social support networks in the welfare system available to employed workers in Norway. For example, in Norway, government-related support includes up to one year of fully paid leave due to illness. These are extremely generous employment conditions that are not offered in many countries. Such increased social and financial support may mean there are minimal external factors affecting employment such as lacking the ability to maintain adequate home/work balance and an increased risk of reduced pay if hours are reduced, relative to both psychological/cognitive barriers and physical barriers. It is interesting nonetheless that scores for external barriers in an Australian cohort of pwMS are very similar (19). By comparison, sickness benefits in Australia are paid by an employer, and depending on the employer, may constitute only 5–10 days per year. Eighty-five percent of Norwegian individuals, however, did not demonstrate this floor effect, indicating that external barriers are still relevant for this population. Compared to a Dutch cohort of pwMS (22), it seems work difficulties in Norway are substantially higher. That could be a reflection of the higher age that was present in the current study compared to the Dutch study (52 vs. 43 years).

The Norwegian version of the MSWDQ-23 showed acceptable construct and concurrent validity. In particular, the MSWDQ-23NV subscales were related to HRQol subscales in the expected direction. The strongest of these relationships were between the psychological/cognitive barriers subscale and the mental health composite score of the MSQOL-54 and between the physical barriers subscale and the physical composite score of the MSQOL-54. Regarding concurrent validity (i.e., being able to distinguish current group or disease status), the MSWDQ-23NV differentiated RRMS from progressive MS types in the physical barriers subscale. Similar levels of difficulties were experienced in the psychological/cognitive domain as well as difficulties relating to external factors. The MSWDQ-23NV differentiated people who worked within the past year vs. those that did not. Considered together this would indicate that all difficulties may predict withdrawal from work, although it is interesting that RRMS experiences psychological and cognitive impact in the workplace just as much as the progressive MS types. Those with RRMS also experience difficulties with external factors. This would suggest that there is a universal need to target psychological/cognitive factors and external barriers in vocational remediation programs.

### 4.1 Strengths and limitations

A clear limitation of the present study is that we did not examine test–retest reliability, which is due to the selected cross-sectional design. However, prior studies have reported a high test–retest reliability for the MSWDQ-23 (21, 23, 28). Thus, there are indications of likely adequate test–retest reliability for the Norwegian version of the MSWDQ-23 but future studies should examine this. Moreover, we did not include healthy controls in our study, which would have helped us attribute the reported work difficulties to MS. Although one of the available MSWDQ-23 validation studies showed good discrimination (27), and notwithstanding the fact that the scale asks participants to report on difficulties that are attributable to their MS, the hypothesis that the reported difficulties in the Norwegian study extend beyond those experienced by healthy individuals was not tested. Finally, this study did not investigate the future employment status of the participants, which would have allowed us to confirm the predictive validity of this instrument. However, previous studies (19, 22) have demonstrated the predictive validity of the instrument for future employment.

A strength of the current study is that we used a confirmatory factor analysis technique to verify the three-factor model structure and evaluated it against both Kline (47) and Hu and Benter (41), documenting a fair fit. Moreover, an advantage of the current study was the inclusion of persons with all types of MS, low EDSS (2.81), and both employed and not employed pwMS. Additionally, the relatively large sample (*n* = 229) can be considered a strength given it is likely to be representative of the Norwegian MS population.

## 5 Conclusion

The Norwegian MSWDQ-23 is an internally consistent and valid instrument to measure perceived work difficulties in persons with all types of MS. The fit of the current data was fair, but the results suggest that the three subscales are strongly related. The MSWDQ-23NV can be considered a useful tool for health care professionals to assess self-reported work difficulties in persons with MS. Thus, it serves to provide tailored follow up to optimize work outcomes. The Norwegian MSWDQ-23 scale should be examined for test-retest reliability and considered implemented in the regular follow up at the MS-outpatient clinics in Norway to support employment maintenance.

## Data Availability

The raw data supporting the conclusions of this article will be made available by the authors, without undue reservation.
