# Supplementary material for: Barriers for work in people with multiple sclerosis: a Norwegian cultural adaptation and validation of the short version of the multiple sclerosis work difficulties questionnaire
Source: Front Rehabil Sci. 2024 Nov 20;5:1404723. doi: 10.3389/fresc.2024.1404723 (PMC11614828; doi:10.3389/fresc.2024.1404723)
Supplement: Supplementary file 1 [file Datasheet1.pdf]

Multiple Sclerosis Work Difficulties Questionnaire - 23 Norwegian Version (MSWDQ-23NV)

INSTRUKSJONER
Personer med MS erfarer ofte vansker på arbeidsplassen som enten er direkte eller indirekte relatert til deres symptomer. De følgende spørsmålene beskriver ulike vanskelige eller problematiske situasjoner som en person med MS kan møte på jobb. Vennligst sett en ring rundt det passende svaret (0, 1, 2..) basert på dine erfaringer i løpet av de siste fire ukene i din nåværende eller din siste jobb. Vennligst svar på alle spørsmålene, og dersom du er usikker på hvilket svar du skal velge, velg det som er nærmest i å beskrive deg.

I løpet av de siste fire ukene, mens du var i din nåværende eller i din siste jobb, vennligst marker hvor ofte du opplevde det følgende som et resultat av din MS.

		Aldri	Sjelden	Noen ganger	Ofte	Nesten alltid
3.	Jeg opplevde manglende koordinasjon av bevegelsene mine	0	1	2	3	4
5.	Jeg syntes at min arbeidsgiver ikke var særlig forståelsesfull for mine behov	0	1	2	3	4
8.	Jeg syntes det var vanskelig å lære noe nytt	0	1	2	3	4
11.	Jeg syntes ikke at min leder eller mine arbeidskolleger var støttende overfor meg	0	1	2	3	4
14.	Jeg følte at forstyrrelser i tarm eller blære distraherte meg fra å gjøre en oppgave	0	1	2	3	4
18.	Jeg trengte en påminnelse for å gjøre en oppgave til en bestemt tid	0	1	2	3	4
20.	Jeg følte at jeg ikke greide å prestere på det nivået som var forventet av meg	0	1	2	3	4
21.	Jeg syntes det var vanskelig å tolerere temperaturen på arbeid	0	1	2	3	4
23.	Jeg syntes det var vanskelig å komme meg til kontoret eller arbeidsstedet mitt	0	1	2	3	4
25.	Jeg strevde med å huske en nylig samtale	0	1	2	3	4
27.	Jeg opplevde smerter mens jeg utførte en oppgave	0	1	2	3	4
28.	Jeg fryktet at jeg ikke ville være i stand til å forsørge meg selv dersom jeg ikke lenger kunne jobbe	0	1	2	3	4
30.	Jeg ble søvnig mens jeg forsøkte å utføre en langvarig oppgave	0	1	2	3	4
33.	Jeg syntes det var vanskelig å holde balansen	0	1	2	3	4
35.	Jeg hadde problemer med å konsentrere meg om en oppgave	0	1	2	3	4
37.	Jeg hadde vansker med å formidle mine tanker til kollegaer	0	1	2	3	4
38.	Jeg følte at det var vanskeligere å balansere jobb og plikter hjemme	0	1	2	3	4
40.	Jeg syntes det var vanskelig å skrive for hånd eller på tastatur	0	1	2	3	4
45.	Jeg syntes det var vanskelig å samhandle med andre	0	1	2	3	4
47.	Jeg fryktet at jeg skulle være inkontinent	0	1	2	3	4
48.	Jeg syntes det var vanskelig å redusere arbeidstiden min fordi da ville lønna mi, nå eller i fremtiden, bli redusert	0	1	2	3	4
49.	Jeg glemte hva min neste oppgave var	0	1	2	3	4
50.	Jeg følte at jobb ble vanskeligere som følge av ansvar hjemme	0	1	2	3	4
